# TAI-SARNET: Deep Transferred Atrous-Inception CNN for Small Samples SAR ATR

**DOI:** 10.3390/s20061724

**Published:** 2020-03-19

**Authors:** Zilu Ying, Chen Xuan, Yikui Zhai, Bing Sun, Jingwen Li, Wenbo Deng, Chaoyun Mai, Faguan Wang, Ruggero Donida Labati, Vincenzo Piuri, Fabio Scotti

**Affiliations:** 1 Department of Intelligent Manufacturing, Wuyi University, Jiangmen 529020, China; ziluy@163.com (Z.Y.); xuanchenoh@163.com (C.X.); wenbodeng92@163.com (W.D.); maichaoyun@foxmail.com (C.M.); faguanhn@163.com (F.W.); 2 School of Electronics and Information Engineering, Beihang University, Beijing 100191, China; bingsun@buaa.edu.cn (B.S.); lijingwen@buaa.edu.cn (J.L.); 3 Departimento di Information, Universita, Degli Studi di Milano, via Celoria 18, 20133 Milano (MI), Italy; ruggero.donida@unimi.it (R.D.L.); vincenzo.piuri@unimi.it (V.P.); fabio.scotti@unimi.it (F.S.)

**Keywords:** Synthetic Aperture Radar (SAR), Convolutional Neural Network (CNN), transfer learning, Atrous-Inception module, lightweight network, small sample

## Abstract

Since Synthetic Aperture Radar (SAR) targets are full of coherent speckle noise, the traditional deep learning models are difficult to effectively extract key features of the targets and share high computational complexity. To solve the problem, an effective lightweight Convolutional Neural Network (CNN) model incorporating transfer learning is proposed for better handling SAR targets recognition tasks. In this work, firstly we propose the Atrous-Inception module, which combines both atrous convolution and inception module to obtain rich global receptive fields, while strictly controlling the parameter amount and realizing lightweight network architecture. Secondly, the transfer learning strategy is used to effectively transfer the prior knowledge of the optical, non-optical, hybrid optical and non-optical domains to the SAR target recognition tasks, thereby improving the model’s recognition performance on small sample SAR target datasets. Finally, the model constructed in this paper is verified to be 97.97% on ten types of MSTAR datasets under standard operating conditions, reaching a mainstream target recognition rate. Meanwhile, the method presented in this paper shows strong robustness and generalization performance on a small number of randomly sampled SAR target datasets.

## 1. Introduction

Synthetic Aperture Radar (SAR) features all-weather, long-range and large-scale detection performance. It can obtain high-resolution radar images under extremely low-visibility weather conditions, effectively identify ground camouflage and masking targets and is widely used in marine environment detection, terrain survey and military target recognition field. SAR Automatic Target Recognition (ATR) is a crucial technique for interpreting SAR target images, which can effectively improve the utilization efficiency of SAR targets images [1]. However, limited by the SAR’s coherent imaging system as well as the electromagnetic scattering mechanism, the SAR target image is full of strong coherent speckle noise, and is affected by the variation of target attitude, angle, imaging parameters and other factors.

Based on the imaging characteristics of SAR target images, researchers have conducted a lot of research on the SAR ATR algorithm. Traditional SAR target recognition methods mainly concentrate on the stage of feature extraction and the construction of a classifier. In the feature extraction phase, Principal Component Analysis (PCA) [2], Independent Component Analysis (ICA) [3], Gray Level Co-occurrence Matrix (GLCM) [4] and Histogram of oriented gradient (HOG) [5] are applied to SAR target recognition tasks. PCA is a multivariate statistical method that examines the correlation between multiple variables. The goal is to extract significant information from the acquired data and then depict it as a new set of orthogonal variables named principal components. Gang et al. [6] put forth a joint multi-channel sparsity method on the basis of robust PCA to improve the display performance of SAR ground moving targets. ICA is an analytical method based on high-order statistical characteristics, which is used to decompose complex datasets into independent sub-parts. Vasile et al. [7] used ICA to the speckle filtering of actual polarized SAR data, which applied the rotational invariant scattering vector derived from each ICA to the minimum mean square error filter, the spatial resolution was better preserved. GLCM is a description of the joint distribution of two gray-level pixels with a certain spatial location relationship, on which the anatomy of the local patterns as well as the arrangement rules of the image is based. Numbisi et al. [8] used random forest and ensemble classifier to average the texture characteristics of SAR target images and the Gray Level Co-occurrence Matrix (GLCM), showing that SAR target images identify cocoa agricultural and transition forests in a multiphase landscape. HOG is a feature descriptor for object detection employed to make calculations of the numerical value of the direction information of the local image gradient. Song et al. [9] designed a HOG for SAR ATR, which accurately captured the target structure in the SAR target image. In terms of classifiers construction, Support Vector Machine (SVM) [10], Adaptive Boosting (Adaboost) [11] and K Nearest Neighbor (KNN) [12] have also been successfully applied to SAR ATR related algorithms. Sukawatanavijit et al. [13] combined genetic algorithm with SVM to present a novel algorithm, which can obtain the optimal classification accuracy of multi-frequency radar satellite-2 (RS2) SAR target images using merely a small number of input features. Kim et al. [14] brought forward a new method for target detection based on Adaboost’s decision-level SAR and IR fusion, which showed satisfactory performance on a synthetic database created by OKTAL-SE. Hou et al. [15] introduced the KNN algorithm to enhance the classification accuracy of super-pixel SAR targets images. This method takes into account the spatial position relationship between pixels and has strong robustness to coherent speckle noise. Eryildirim et al. [16] proposed a novel method for extracting descriptive feature parameters from two-dimensional cepstrum of SAR images, which had a lower computational cost than PCA. Clemente et al. [17] utilized pseudo-Zernike moments of multi-channel SAR images as features to identify different targets, and realized high confidence ATR. Sun et al. [18] introduced a SAR images recognition method based on dictionary learning and joint dynamic sparse representation, which accelerated the recognition speed and accuracy. Clemente et al. [19] designed a SAR ATR algorithm based on Krawtchouk moments, which had strong target classification ability and anti-noise ability. However, the above algorithms heavily rely on cumbersome manual feature design and empirical selection, which is not only costly, but also the generalization ability of the designed model is often poor.

CNN is capable of processing multi-dimensional data and has powerful representation learning capabilities, therefore having attracted the attention of many researchers. Since AlexNet [20] won ImageNet Challenge: ILSVRC 2012 [21] and demonstrated the power of CNN, CNN has begun to appear in various computer vision tasks. Then VGG Net [22] used a sequential structure to explore the impact of CNN depth on image classification tasks, showing that the depth of the network has greatly contributed to the excellent algorithm, and achieved the second place in ILSVRC 2014. ResNet [23] introduced the concept of residual representation into the construction of CNN models, which further extended the depth of CNN and achieved better performance. GoogLeNet [24] provided another idea for the design of CNN models, which proposed the Inception module that greatly improved the utilization of parameters by expanding the width of the network and using smaller convolution kernels. In recent years, various models based on CNN have made remarkable achievements in the optical image target recognition tasks. SAR target image recognition tasks have been stimulated by this and plenty of related research has been carried out. Chierchia et al. [25] adopted a residual learning strategy in the designed CNN to denoise the SAR target images by subtracting the recovered speckle component from the noise component. Pei et al. [26] augmented the data by generating sufficient multi-view SAR data and fed the expanded SAR data into a designed CNN with a multi-input parallel network topology for identification. Dong et al. [27] utilized spatial polarization information and XGBoost to perform classification experiments on the PolSAR images of the Gaofen-3 satellite, and proved that the combination of spatial information helps improve the overall performance. Wang et al. [28] proposed a fixed-feature-size CNN, which realized the classification of all pixels of a PolSAR image at the same time, and improved the classification accuracy by using the correlation between different land covers. Shao et al. [29] effectively reduced the impact of data imbalance on SAR image recognition results by introducing a visual attention mechanism and a new weighted distance measure loss function into the designed network. Zhang et al. [30] improved the scattering decomposition technology based on the multi-component model and adopted a superpixel-level classification strategy for the extracted multiple features, providing a new method for land use classification of PolSAR data. He et al. [31] designed a special generative adversarial network to generate enough labeled SAR data, which improved the classification performance of CNN on SAR images. Although the CNN-based SAR ATR algorithm has achieved breakthroughs in recognition performance, there are still three crucial problems in the field of SAR target image recognition that need to be resolved. First, the SAR target images are full of coherent speckle noise, resulting in highly redundant training sample features, lack of representative features for target recognition and greatly affecting the classification performance of the model. Second, limited by the expert domain knowledge and labeling costs of SAR target images, labeled SAR data is scarce. Training a CNN with a small amount of sample SAR data will cause serious overfitting and the model generalization ability is poor. Third, deep CNNs have complex structures and enormous computational complexity, which are not conducive to the development of terminal equipment for SAR identification systems.

In view of the above challenges, a lightweight network architecture TAI-SARNET combined with transfer learning is put forward here to achieve efficient SAR target image recognition. Firstly, the Atrous-Inception module with a small size convolution kernel is adopted in the network to increase both the depth and width of the network while increasing the receptive field and reducing the number of parameters. Secondly, Batch Normalization (BN) [32] is employed behind each convolutional layer to effectively prevent network overfitting, and Global Average Pooling [33] is exploited to further decrease the number of parameters. Subsequently, the robustness of the proposed algorithm on a small amount of sample data is verified, and transfer learning is introduced to enhance the performance of the model. Eventually, the suggested algorithm is tested on the MSTAR [34] database, and the experimental results have demonstrated that it attains excellent recognition accuracy. The main contributions of this paper are summarized as follows:

(1) An improved lightweight CNN model based on the Atrous convolution and Inception module is proposed. This model can obtain a rich global receptive field, effectively preventing the network from overfitting, and obtain high recognition accuracy on the MSTAR dataset.
(2) The Atrous-Inception module is designed to extract more detailed target feature information, and it has strong robustness on a small sample dataset of the constructed SAR targets images.
(3) The transfer learning strategy is used to explore the performance of the prior knowledge based on optical, non-optical, hybrid optical and non-optical fields transferred to the SAR targets images recognition tasks, further improving the robustness as well as the generalization of the model on the SAR small sample datasets.

The main work arrangement of this paper is as follows: The second part introduces the related work of CNN and transfer learning. The third part introduces the methods in this paper. The fourth part introduces the experimental results and analyzes them. The fifth part draws the conclusion.

## 2. Related Work

### 2.1. CNN

CNN is a feed-forward neural network with a deep structure that contains one or more convolution operations, enabling the transformation of mapping input to output. Compared with other neural networks, CNN’s most significant feature is the introduction of the concept of local connection and parameter sharing. Local connection can greatly reduce the amount of network model parameters, speed up model convergence and reduce the need for computing hardware. Parameter sharing refers to the convolution kernel with the same parameters to extract the features on the image multiple times, and the extracted features are combined non-linearly, so that the network can automatically extract better features. The above characteristics break through the limitations of traditional machine learning algorithms based on artificial design features and are widely used in many fields.

GoogleNet marks a significant milestone in the history of CNN. It first proposes the Inception structure, which performs convolution and pooling operations upon input images in parallel and increases the adaptability of the network to multiple scales. At the same time, GoogleNet adopts the bottleneck layer design in the Inception module to achieve feature reduction, improves the local perception area of CNN and reduces the calculation of network parameters. Subsequently, Inception-v2 is proposed based on GoogleNet, and BN is introduced. On the one hand, the BN layer solves the problem of internal covariate shift and speeds up training. On the other hand, the BN layer plays the role of regularization, which effectively prevents the phenomenon of network overfitting. After that, Inception-v3 [35] introduces the concept of factorization, using asymmetric convolution to build Inception modules. Specifically, the convolution kernel of size N × N is decomposed into convolution kernels of size N × 1 and 1 × N, effectively reducing the number of network parameters while achieving the same effect. Inception-v4 [36] further combs the Inception series and introduces a stem module to build a deeper network structure. In addition, inspired by ResNet, the researchers combined the Inception module with the residual structure to propose Inception-ResNet, which further reduced the rate of image recognition errors. Shao et al. [37] tested the ability of the Inception-v3 network for SAR targets image recognition tasks and achieved good recognition results. Chen et al. [38] realized the high-precision detection of small and dense ships in SAR images by adding the Inception module and residual structure to the designed network architecture. Zhang et al. [39] used GoogleNet to classify Gaofen-3 PolSAR images and achieved good results on SAR target image tasks with different sampling intervals. Wang et al. [40] developed an automatic classifier of S-1 WV marine SAR target images based on the Inception-v3 structure, showing great potential in marine SAR scene recognition.

### 2.2. Transfer Learning

Traditional machine learning algorithms presume that training data and testing data follow the same data characteristic spatial and data characteristic distribution, but this assumption usually fails to meet the actual application scenario. In some specific areas, where data accessibility and labeling costs are difficult to obtain sufficient training data, transfer learning provides a solution to this situation [41]. It starts by obtaining a large amount of easily accessible data from relevant domains and then uses that data to create high-performance learners. Those high-performance learners obtain prior knowledge from previous tasks and apply the acquired knowledge to the current tasks in a feasible way. In a nutshell, transfer learning is used to advance learners in another field by acquiring relevant domain information. Therefore, it is a feasible method to obtain the labeled data in relevant fields according to the actual situation and use transfer learning to enhance the accuracy of SAR target recognition tasks. In addition, the neural network can retain the skills learned from the original tasks, and continuous learning can be achieved through transfer learning, which improves the generalization of the model.

Transfer learning breaks through the limitations of traditional machine learning algorithms, allowing the task or data distribution of the source domain as well as the target domain to differ greatly, and effectively alleviate the lack of labeled data for the target tasks. The advantages of transfer learning are obvious, and researchers have also applied transfer learning to SAR target recognition tasks. Huang et al. [42] uses a plenty of unlabeled SAR scene images to train the superimposed convolutional autoencoder and then transfers the acquired knowledge to the task of SAR target image classification with limited amounts of labels, which effectively solves the problem of failing to train deep CNN because of limited amount of labeled SAR data. David et al. [43] pioneers in the study of transfer learning in simulated datasets and SAR real datasets, showing that prior knowledge of transferring simulation data can effectively speed up the convergence of models and improve the accuracy of SAR target image recognition. Zhong et al. [44] propose a SAR target image classification method based on transfer learning and model compression, which effectively solves the training deep CNN overfitting phenomenon caused by sparse SAR data. Rostami et al. [45] propose a method for classification of small samples based on deep CNN. This method transfers Electro-Optical domain knowledge to the SAR target image classification tasks by obtaining easy-to-access labeled data from the relevant Electro-Optical domain, and then learning from a shared, unchanged cross-domain space. It is proved Electro-Optical domain knowledge can be very good migration to the SAR domain of ship classification tasks.

## 3. Proposed Methods

In this section, we propose a lightweight SAR target image classification network combining the atrous convolution and the Inception module, and the application of transfer learning is introduced in this paper. The first part displays the basic network architecture. The second part illustrates the Atrous-Inception module in detail and elaborates on the related concepts of the receptive fields. The third part derives the mathematical formula of the design principle of the optimization algorithm used in this paper. In the fourth part, we give the specific definition of transfer learning and introduce the transfer learning strategy adopted in this paper.

### 3.1. Proposed Network

**Figure 1:**
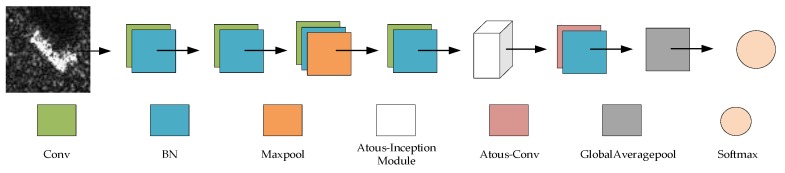
The structure of the proposed network.

This paper presents a lightweight network based on atrous convolution and Inception-v3, which we call TAI-SARNET, the specific network structure is presented in Figure 1, and the detailed parameter information of the structure is shown in Table 1.

The first four layers of TAI-SARNET are consistent with Inception-v3, and output 80 feature maps after passing the first four layers of the network. Then, an Atrous-Inception module is immediately followed, which contains three atrous convolution layers and outputs 96 map features, and details of The Atrous-Inception module will be introduced in Section 3.2. Finally, an atrous convolution layer is placed behind the Atrous-Inception module, which outputs 256 feature maps. The above structure constitutes the feature extraction part of TAI-SARNET. In particular, it should be noted that the activation function used in TAI-SARNET is ReLU and the BN layer is added behind each convolution layer. The addition of the BN layer can dramatically accelerate the speed of network training, effectively address the problem of gradient dispersion and avoid overfitting of the network. The formula of the BN layer can be expressed as follows:

(1) $$y_{i}=\alpha \left(\frac{x_{i}-\mu_{B}}{\sqrt{\sigma_{B}^{2}+\varepsilon }}\right)+\beta$$

where $x_{i}$ represents the i-th input of the BN layer, $\mu_{B}$ is the mean of a batch input data, $\sigma_{B}$ represents the standard deviation of the batch input data and ε is a very small constant value. α and β are learnable reconstruction parameters, which are iteratively optimized through network training. By Equation (1), the i-th output $y_{i}$ of the BN layer can be calculated.

Subsequently, the global average pooling layer is utilized in place of the traditional fully connected layer so as to realize the calculation of all pixel averages for each feature map output from the feature extraction part. The global average pooling layer is employed to directly reduce the data dimension, greatly reducing network parameters and play the role of regularization in the entire structure to prevent network overfitting. Finally, the data values from the global average pooling layer are input into the classifier softmax. The essence of softmax is a normalized exponential function that is functionally normalized as the probability distribution value within the 0–1 interval, and the formula of the softmax layer can be represented as follows:

(2) $$soft\max\left(y_{p}\right)=\frac{e^{y_{p}}}{\sum_{q=1}^{n}e^{y_{q}}}$$

In Equation (2), $y_{p}$ represents the p-th output value of the global average pooling layer, and n represents the number of categories. After softmax normalization, each output value can be regarded as the probability value of identifying the target for that category, and the sum of the output values for all categories of the network is one.

### 3.2. Atrous-Inception Module

The receptive field is used to represent the receptive range of different neurons in the network to the original image. The larger the value of neuron receptive field is, the greater the range of the original image it can access, which means that it contains more global and higher-level semantic features. In traditional CNNs, a convolution operation is first used to extract feature maps, and then downsampling is performed by a pooling operation to increase the receptive field. However, this operation can result in the loss of the internal structure and spatial information of the target, and the information of the small target object can not be reconstructed. Atrous convolution [46] is a good solution to the problems caused by frequent use of pooled operation, and is widely used in the field of semantic segmentation of images. Specifically, the atrous convolution inserts holes in the standard convolution, which ensures that the receptive field is increased without losing image information, enabling each convolution output to contain a greater range of information.

The size of the receptive field of the standard convolution is pertinent to the size of the current layer of the convolution kernel, the moving step of the convolution kernel, and the size of the receptive field of the previous standard convolution. The receptive field calculation formula for standard convolution is defined as follows:

(3) $$r_{n}=r_{n-1}*k_{n}-\left(r_{n-1}-\prod_{i=1}^{n-1}s_{i}\right)*\left(k_{n}-1\right),\;n\geq 2$$

where $r_{n}$ in the formula represents the receptive field in the n-th convolution layer, $k_{n}$ represents the size of the convolution kernel in the n-th convolution layer, $s_{i}$ indicates the moving step size of the convolution kernel in the i-th convolution layer and the symbol ∗ represents the multiplication of two factors. In particular, the size of the receptive field of the first convolution layer is the size of the convolution kernel of the convolution layer. Simplify the formula as follows:

(4) $$r_{n}=r_{n-1}+\left(k_{n}-1\right)*\prod_{i=1}^{n-1}s_{i},\;n\geq 2$$

For example, if the first layer is a 3 ∗ 3 standard convolution, then the receptive field of this convolution layer is 3 ∗ 3. After the first layer of standard convolution is superimposed with a 3 ∗ 3 standard convolution, then the receptive field is 5 ∗ 5. If three 3 ∗ 3 standard convolutions are superimposed, the receptive field is 7 ∗ 7.

Compared to the standard convolution, the atrous convolution has a hyperparameter called dilated rate, which refers to the number of intervals between convolution kernels. The calculation formula of the receptive field of the hollow convolution is as follows:

(5) $$r_{n}=r_{n-1}+\left(k_{n}-1\right)*d_{n}*\prod_{i=1}^{n-1}s_{i},\;n\geq 2$$

**Figure 2:**
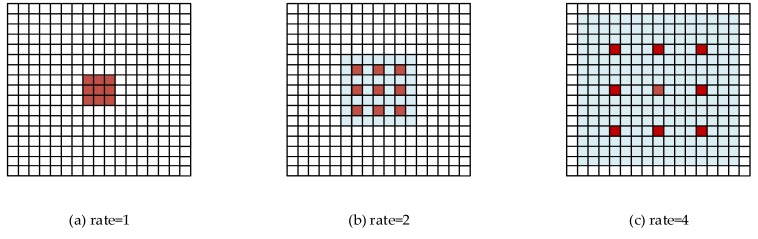
Atrous Convolution.

The Equation (5) represents the dilated rate in the first atrous convolution layer, and the atrous convolution of the dilated rate of 1 is equivalent to a standard convolution. Figure 2 shows that the receptive field after 3 ∗ 3 atrous convolution with the dilated rate of 1, 2 and 4 have reached 15 ∗ 15, which indicates that in the same network depth, the receptive field of the atrous convolution is much larger than the standard convolutional.

**Figure 3:**
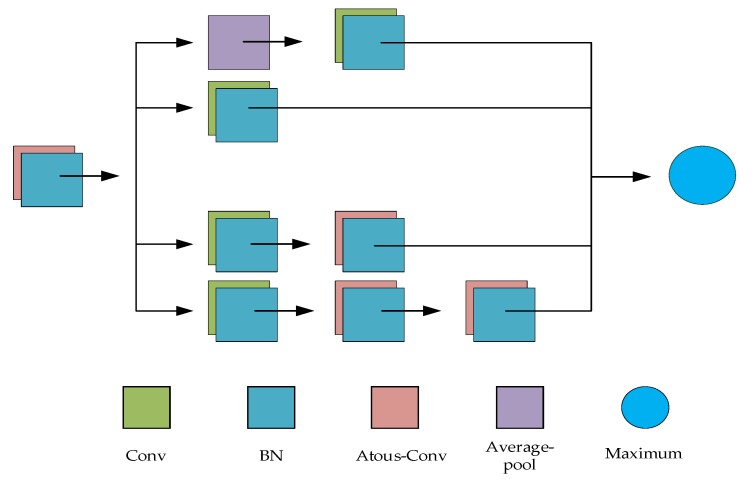
Structure of the Atrous-Inception module.

In the Inception series of networks, the strategy adopted is to use a small convolution kernel stack rather than a large convolution kernel, which is able to reduce the parameters while obtaining the receptive field of the same size as the large convolution kernel. However, expanding the receptive field by superimposing a small convolution kernel can only grow linearly, while atrous convolution can increase the receptive field exponentially without increasing the parameter amount. Therefore, the Atrous-Inception module is used to grow the receptive field, obtain a larger range of spatial information, and further control the number of network parameters. The detailed structure of the Atrous-Inception module is displayed in Figure 3, the first part uses an atrous convolution layer with dilated rate of 4 and a convolution kernel size of 3 ∗ 3, the second part uses an improved Inception module. In this improved Inception module, we maintain the main structure of the original Inception, using four 1 ∗ 1 standard convolutions to build the bottleneck layer to achieve feature reduction, and using average pooling to decrease the number of parameters. On top of the original Inception structure, we replace the three 3 ∗ 3 standard convolutions on the two branches with the dilated rate of 4 and convolution kernel size of 3 ∗ 3, which can increase the receptive field of the network and obtain more global information. In the final merge operation, the Maximum merge method is adopted, which realizes the output element-wise maximum corresponding to the feature map. Compared with the original Inception structure using Concatenate merge, Maximum avoids the increase of feature dimensions and greatly reduces the number of parameters.

### 3.3. RMSProp Optimization

The gradient descent algorithm is a typical optimization method commonly used in CNNs, and the core of the backpropagation algorithm is to continuously use gradient descent to update the weight parameters of each layer for optimization. However, the gradient descent algorithm needs to traverse all samples per iteration, making the training process slow and taking a long time to reach convergence. The RMSProp algorithm is an adaptive learning rate algorithm propounded by Hinton [47], which uses the exponential decay average of historical gradients and discards the gradients at the earlier points, thus speeding up the convergence speed of the algorithm. The related calculation formula of RMSProp vector update is shown in Equation (6):

(6) $$E{\left[g^{2}\right]}_{t}=\gamma E{\left[g^{2}\right]}_{t-1}+\left(1-\gamma \right)g_{t}^{2}$$

In Equation (6), $g_{t}$ represents the gradient of the current moment, γ is a manually set fractional value and $E{\left[g^{2}\right]}_{t}$ represents the running average of the attenuation of the square gradient at the time step t. It can be known from the formula that $E{\left[g^{2}\right]}_{t}$ depends only on the current gradient and the previous gradient average.

The calculation formula for parameter update is as follows:

(7) $$\theta_{t+1}=\theta_{t}-\frac{\eta }{\sqrt{E{\left[g^{2}\right]}_{t}+\varepsilon }}g_{t}$$

In Equation (7), $\theta_{t}$ represents the parameter at the time step t, η represents the initial learning rate and ε represents a smoothing term that avoids the denominator of 0, in which ε generally takes 1 × 10^−8^. The RMSProp algorithm is applied to update according to the parameter update rule of the above formula, one of the optimization learning algorithms commonly used in deep learning.

### 3.4. Transfer Learning

When the distribution of training data and testing data does not meet the prerequisites of the same distribution, traditional machine learning algorithms cannot achieve satisfactory performance. However, due to the cost of manual annotation and data accessibility, it is difficult to construct a qualified dataset from scratch. Transfer learning is capable of improving the learning effect of related new tasks through transferring knowledge from already learned tasks, breaking through the limitations of data distribution differences and the lack of a vast quantity of labeled data in the related target domain. Specifically, transfer learning transfers the knowledge learned from a source domain incorporating a large number of labeled training samples to a related target domain with a small number of labeled samples, so that the target domain achieves better results. In addition, transfer learning can reuse previous models, thus greatly speeding up learning efficiency. For more rigorous expressions, the relevant definitions and symbols of transfer learning are as follows: Given a domain D={χ,P(X)}, where χ represents the feature space, P(X) represents the edge probability distribution and $X=\left\{x_{1},x_{2},\ldots ,x_{n}\right\}\in \chi$. Then given a task T={ϒ,f(⋅)} corresponding to the domain D, where ϒ represents the label space and f(⋅) represents the target prediction function. The training set data pair can be represented as $\left\{x_{i},y_{i}\right\}$, where $x_{i}\in X$, $y_{i}\in ϒ$. f(x) represents the label value of the unlabeled test sample x. In transfer learning, the domain that has been learned is called the source domain $D_{S}=\left\{\chi_{S},P\left(X_{S}\right)\right\}$, and the related domain to be improved is called the target domain $D_{T}=\left\{\chi_{T},P\left(X_{T}\right)\right\}$. The task corresponding to the source domain $D_{S}$ is called the source task $T_{S}=\left\{{ϒ}_{S},f_{S}\left(\cdot \right)\right\}$, and the task corresponding to the target domain $D_{T}$ is called the target task $T_{T}=\left\{{ϒ}_{T},f_{T}\left(\cdot \right)\right\}$. The goal of transfer learning is to use the knowledge learned from the source domain $D_{S}$ and the source task $T_{S}$ to improve the prediction capability of the prediction function $f_{T}\left(\cdot \right)$ in the target domain $D_{T}$, and require $D_{S}\neq D_{T}$ or $T_{S}\neq T_{T}$. SAR targets images are typical non-optical images, this paper explores the performance of transferring prior knowledge from the optical, non-optical, and hybrid optical and non-optical domains to SAR targets recognition tasks.

**Figure 4:**
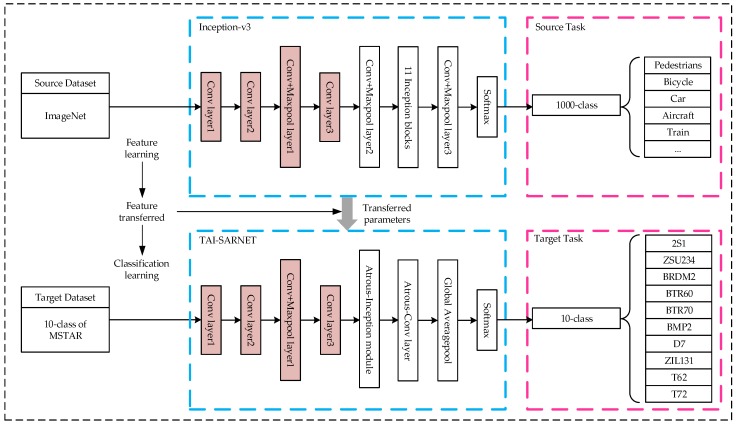
Transfer prior knowledge of optical images.

For the optical domains, the source domain $D_{S}$ dataset uses the ImageNet, the source task $T_{S}$ is classified into 1000 classes of optical target images, the target domain $D_{T}$ dataset uses the 10-class MSTAR dataset under Standard Operating Condition (SOC), and the target task $T_{T}$ is 10 classes of SAR target image classification. The features extracted by shallow networks are more general, therefore the structure of the first four layers designed by TAI-SARNET is the same as that of the first four layers of Inception-v3, and the transfer based on the parameter migration of specific layers is used. Specifically, first obtain the Inception-v3 model pre-trained on $D_{S}$, extract its underlying parameters and transfer to the corresponding layer of TAI-SARNET used in $D_{T}$ and then retrain the entire network until the model reaches a convergence state and obtains the optimal result. The specific transfer process is shown in Figure 4.

**Figure 5:**
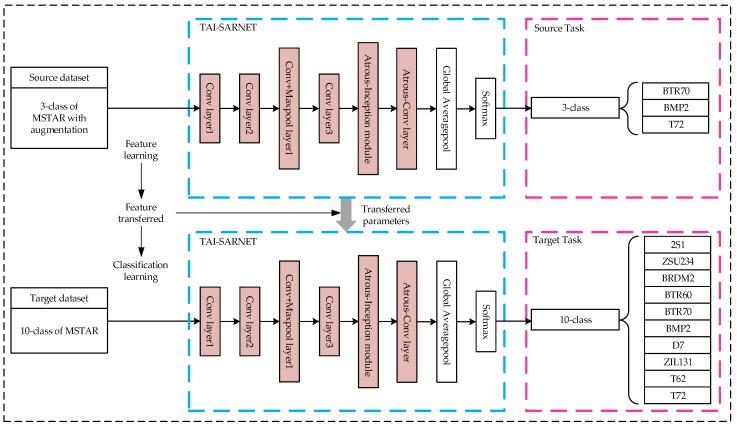
Transfer prior knowledge of non-optical images.

For the non-optical domains, the source domain $D_{S}$ dataset uses the 3-class MSTAR dataset with augmentation, the source task $T_{S}$ is 3 categories of SAR target image classification, the target domain $D_{T}$ dataset uses 10-class MSTAR dataset under Standard Operating Conditions (SOC) and $T_{T}$ is the classification of 10 categories of SAR target images. First, we use the full-angle rotation enhancement method to augment the 3-class MSTAR dataset by 360 times as the $D_{S}$ dataset and then use the enhanced data for $T_{S}$ to train on TAI-SARNET to obtain a pre-trained model. Finally, the network on $D_{T}$ loads the specific layer weights of the pre-trained model and fine-tunes the training by changing the classification number of the softmax layer according to $T_{T}$ until the model reaches a convergent state and $f_{T}\left(\cdot \right)$ obtains the optimal prediction result. The specific transfer process is shown in Figure 5.

For the purpose of fully comparing the transfer effects of data from different imaging modes as source domain knowledge, we take the classification task of combining optical and non-optical radar images as source task $T_{S}$ and transfer the knowledge obtained from $T_{S}$ to the 10 classes of SAR targets images classification which is target task $T_{T}$. Specifically, the NWPU-RESISC45 dataset [48] and the 3-class MSTAR dataset extended by random angle rotation are mixed to construct a complete hybrid radar image dataset as the source domain $D_{S}$ dataset. The number of the training set for the complete hybrid radar dataset is 23,472, the number of the validation set is 6768, and the number of the testing set is 4515. The transfer strategy is consistent with Figure 5, starting with training at TAI-SARNET in the hybrid radar image to obtain a pre-training model. Then, keep the feature extraction part of TAI-SARNET and adjust the classifier based on $T_{T}$ only. Finally, the weights of the pre-trained model are fine-tuned to the network of target domain A and retrained until the model converges on the 10-class MSTAR dataset under SOC.

## 4. Experimental Results and Analysis

### 4.1. Dataset

**Figure 6:**
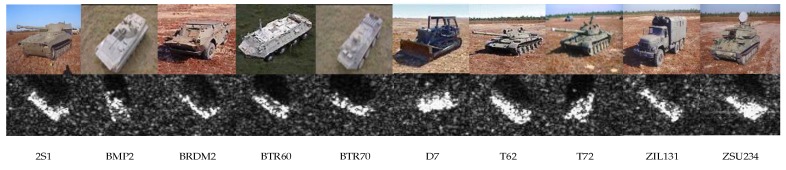
MSTAR dataset image example.

In order to demonstrate the performance of the algorithm on SAR target recognition tasks, unified experimental verification is performed using the MSTAR dataset. The MSTAR dataset is the measured SAR ground stationary target data released by the MSTAR program and supported by the US Defense Advanced Research Projects Agency, which contains SAR target images obtained at various azimuths by multiple vehicle targets. The MSTAR dataset contains ten types of ground targets under Standard Operating Condition (SOC), including artillery (2S1, ZSU234), armored vehicle (BRDM2, BTR60, BTR70, BMP2, D7, ZIL131) and tank (T62, T72), where BRDM2 in the armored vehicle category contains three types of variants (9563, 9566, C21), T72 in the tank category contains three types of variants (132, 812, S7). The SAR target image resolution is 0.3 m × 0.3 m and the pixel size is 128 × 128, and the optical target image and the corresponding SAR targets image are presented in Figure 6. In particular, the depression angles of the training set and testing set images in the 10-classes MSTAR dataset under SOC are 17° and 15°, respectively. The BMP2 in the training set contains only the 9563 series and the T72 contains only the 132 series, while BMP2 and T72 in the testing set contain all three types of variants. So as to adapt to the network structure proposed in this paper, the pixel size of the image after ROI extraction is 64 × 64, and the specific SOC 10-classes MSTAR dataset configuration is shown in Table 2.

### 4.2. Experimental Environment and Configuration

All experiments in this paper were run on a computer configured with AMD Ryzen 5 2600X processor, Nvidia GeForce RTX 2080 (8 GB) GPU and 32 GB RAM. The compilation environment of all experiments is unified to Ubuntu 18.04 system, and Keras deep learning framework developed by TensorFlow as the backend is used for training.

### 4.3. Training/Validation Strategy

The optimizer used in this paper is RMSprop, the initial learning rate η is set to 1 × 10^−4^, and 250 epochs are trained. The learning rate decay strategy is used during the experiment. The specific settings are:

Monitoring indicators: validation loss, learning rate attenuation factor: factor = 0.5, waiting for transition rounds: patience = 20. When the monitored validation loss has not decreased after the number of patience rounds, the learning rate attenuation strategy will be triggered and the learning rate will be reset using Equation (8):

(8) new_lr=η∗factor

**Figure 7:**
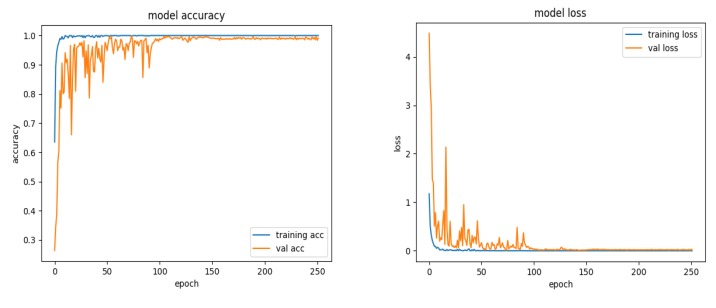
Visualization graphs for training and validation.

The complete experimental process is expressed as follows: First, the image size of the MSTAR dataset under SOC is changed to 64 ∗ 64 through ROI extraction, and the complete training set is re-divided into a training set and a validation set in accordance with the ratio of 75% and 25%. Then, the training set data is fed into TAI-SARNET for training, and the validation set is used for real-time verification. Finally, the trained model is tested on the testing set to get the final recognition result. As it is shown in Figure 7, the whole training and validation curves are visualized. By monitoring the visualized curves, the model fitting state in the training process can be dynamically understood, so as to adjust the relevant training strategies. The curves of training accuracy and validation accuracy almost coincide at the 150th epoch and are close to 100%. Moreover, the curves of training and validation have no obvious fluctuation in the subsequent 100 epoch, indicating that the model has reached the convergence state. On the other hand, the training loss and the validation loss almost coincide at the beginning of the 100th epoch, and there is no obvious fluctuation in the subsequent training process, indicating that the results of the training and validation are highly fit.

### 4.4. Analysis of the Proposed TAI-SARNET

In this section, in order to explore the optimal network settings, different structures of TAI-SARNET will be tested on the dataset configuration mentioned in Section 4.1.

#### 4.4.1. Evaluation on Various Dilated Rate

The dilated rate is one of the key factors exerting an impact on the receptive field of the atrous convolution layer, and it makes sense to explore the performance effect of different dilated rates on TAI-SARNET. As shown in Table 3, TAI-SARNET configured with four types of dilated rates are tested. When the dilated rate is 1, the atrous convolution layer is equivalent to the standard convolution layer, and the recognition accuracy is 91.72%. When the dilated rate is increased to 2, the recognition accuracy is improved by 3.84% compared to the standard convolution layer, which shows that using the atrous convolution layer is more effective than the standard convolution layer in SAR targets recognition tasks. When the dilated rate is set to 4, the recognition accuracy reaches a maximum of 97.97%, which is 2.41% higher than the atrous convolution layer with a dilated rate of 2. When the dilated rate is further increased to 6, the recognition accuracy rate is only 88.63%, compared with the standard convolution layer, it decreases by 3.09%, compared with the highest recognition accuracy rate, it even decreases to 9.34%, which indicates the setting of the dilated rate is not the bigger is the better. Constrained by the network structure, continuing to expand the dilated rate to 8 will result in negative feature dimensions, so the dilated rate of the final TAI-SARNET is set to 4.

#### 4.4.2. Evaluation on Various Atrous-Inception Module

The test results of TAI-SARNET embedded with different numbers of Atrous-Inception modules on the MSTAR dataset are shown in Table 4. When the Atrous-Inception module is not added to the network, the total parameters of the network are low, but the recognition accuracy is only 94.28%. When an Atrous-Inception module is added, the recognition accuracy reaches 97.97%, and the total number of parameters is still at a low level. When two Atrous-Inception modules are added, the recognition accuracy is 1.66% lower than when only one Atrous-Inception module is added, and the total number of parameters also grows significantly. When the quantity of Atrous-Inception module is increased to three, although the recognition accuracy rate is increased by 1.31% compared with the case without adding Atrous-Inception modules, the total number of parameters increases by nearly 7 folds. The lightweight network architecture requires comprehensive consideration of model parameters and recognition accuracy, so only an Atrous-Inception module is added to TAI-SARNET.

#### 4.4.3. Evaluation on Various Merge Ways

The operation objects of the merge layer are chiefly divided into two categories, one is to operate on the channels of the feature maps, and the other is to operate on the elements in the feature maps. The merge layer mode for channel operation is Concatenate, which connects multiple feature maps to be merged, eventually increasing the number of channels on the feature maps, but the amount of information on each feature map does not increase. The merge layer modes for elements operations include Add, Multiply, Average, Minimum and Maximum, and these operations are premised on ensuring that the amount of feature maps channels of the merge layer to be fused is consistent. By operating the feature maps element by element, it does not increase the number of channels of the feature maps, thus controls the growth of the parameter amount. As shown in Table 5, Maximum achieves the best result among all merge layer modes and has nearly half the amount of parameters compared to Concatenate based on channel operation. Therefore, the Maximum merge operation is used in the network in this paper.

#### 4.4.4. Evaluation on BN

The BN layer can effectively alleviate the overfitting phenomenon during the network training process. To explore the actual effect of the BN layer in TAI-SARNET, ablation experiments are performed. The experimental results are demonstrated in Table 6, the recognition accuracy of the BN layer added after each layer of convolution is 97.97%, while that without the BN layer is only 89.60%. This indicates that the BN layer performs a certain role in improving the recognition accuracy of the model.

#### 4.4.5. Confusion Matrix

Table 7 is the confusion matrix of the test results of the 10-class MSTAR dataset under SOC based on the proposed method. So as to clearly show the test results of the proposed method on each type of target, we use different color patches to label the classification of the three advanced categories. Yellow color block indicates artillery, blue color block indicates armored vehicles, green color block indicates tanks and black bold figures on the diagonal of each color block indicate the number of correctly identified targets for each subclass in the advanced category. The bold red figures in each color block indicate the number of subclass targets in the same advanced category that has been misidentified as targets in other subclasses. The bold red figures outside each color block indicate the number of targets that have been misidentified as targets in other advanced categories. It can be seen from Table 7 that the artillery and tank classes do not have red bold figures in their respective color blocks, only a small number of red bold figures appear outside the color blocks, and the identification accuracy of the two advanced subclasses are very high, artillery sub-class 2S1 and tank sub-class T62 even reach 100% recognition accuracy, indicating that our proposed method has high recognition accuracy in these two advanced classes. In the blue color block of the armored vehicle class, we find that the subclasses BRDM2, BTR70, D7 and ZIL131 all have high recognition accuracy, but the subclasses BTR60 and BMP2 have low recognition accuracy. Among them, the armored vehicle subclass BTR60 is misclassified as a subclass BTR70, which may be engendered by the BTR70 being an upgraded version of the BTR60. The armored vehicle subclass BMP2 is misclassified into some other advanced categories. This may be the 9563 model that only contains BMP2 in the training set under SOC, but the testing set contains three variant series including 9563, 9566 and C21. However, the BMP2 recognition accuracy also reaches 95.06%. It is worth noting that the T72 in the training set contains only 132 models, but the testing set contains 132, 812 and S7 variants, and its recognition accuracy reaches 98.63%, showing that the proposed network acquires certain ability to identify variants. Finally, the proposed network reaches 97.97% in overall recognition accuracy.

### 4.5. Robustness Evaluation on Small Samples Dataset

The CNN-based model relies on a sufficient number of labeled training samples and achieves good recognition results. However, it is arduous to obtain a large amount of labeled data in the SAR scene, and the recognition results of CNN on small samples datasets of SAR are not ideal. In addition, the SAR target images are full of coherent speckle noise, thus making it difficult for CNN to extract key features and resulting in poor robustness of the model. In view of the above problems, we will explore the robustness of the network proposed in this paper on a small sample dataset of SAR in this section.

**Figure 8:**
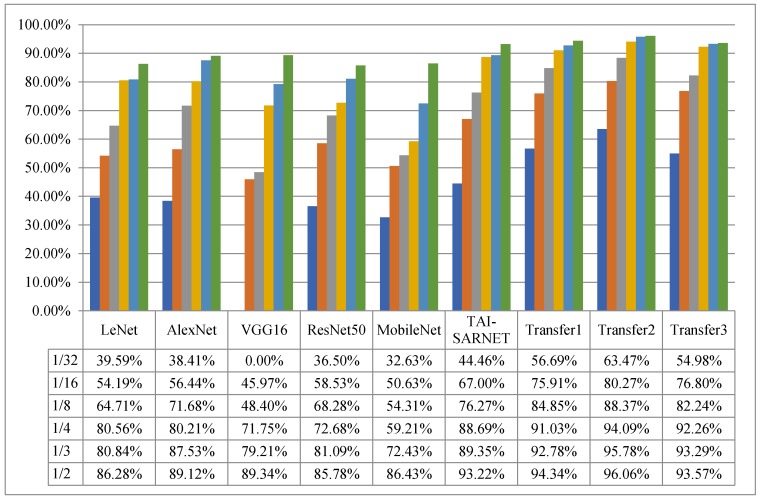
Result on state-of-the-art model on small samples.

ResNet50, MobileNet and TAI-SARNET are verified on small sample datasets constructed, and the recognition accuracy of each network is presented in Figure 8. The experimental results have shown that the recognition accuracy of TAI-SARNET proposed in this paper on all small samples datasets significantly exceeds that of many classic networks that perform well in optical images classification tasks. As the number of samples decreases to 1/32 of the original data, VGG16Net cannot converge. Other classical networks need to increase the amount of iterations to ensure that the model can converge, while our network still converges without increasing the number of iterations. From the above analysis, we can see that when the number of training samples are very limited, our method has obvious advantages, indicating that the method has favorable robustness on small samples datasets of SAR.

### 4.6. Transfer Learning on Small Samples dataset

To explore the performance of transferring images of different imaging patterns as source domain knowledge for the domain of SAR targets, we have verified on small samples datasets established in Section 4.5. We describe transfer methods based on the knowledge of the optical domain, non-optical domain, and combined optical and non-optical domains as Transfer1, Transfer2 and Transfer3 respectively, and compare them with other advanced methods. The experimental results are shown in Figure 8.

#### 4.6.1. Transferring Prior Knowledge of Optical Images

First, the source domain dataset uses the optical dataset ImageNet and pre-trained the model on Inception-v3. Secondly, the target domain dataset uses 10-class MSTAR dataset under SOC, and then uses the parameter-based transfer method to load the underlying weights onto the TAI-SARNET presented in this paper, the recognition results are shown as Transfer1 in Figure 8. Compared with the model trained from scratch, the performance of the method by transferring knowledge in the optical domain has improved by 1.12%, 3.43%, 2.34%, 8.58%, 8.91%, 12.23% on the small samples dataset of 1/2, 1/3, 1/4, 1/8, 1/16 and 1/32, respectively. From the experimental results shown above, it can be known that transferring the low-level parameters of the pre-trained network of a large optical dataset is helpful for SAR targets recognition tasks.

#### 4.6.2. Transferring Prior Knowledge of Non-Optical Images

To explore the impact of non-optical images knowledge as a source domain on the SAR targets images recognition task, we use 3-class MSTAR dataset (one of the configurations of the MSTAR dataset, which includes only T72, BMP2 and BTR70) after 360° rotation enhancement as the source domain dataset, and 10-class MSTAR dataset under SOC is used as target domain dataset. First, the TAI-SARNET is used to train on the 3-class MSTAR dataset with full-angle rotation augmentation to obtain a pre-trained model. Then, we load the weights of the pre-trained model on the target task for fine-tuning. As shown in Transfer 2 in Figure 8, the recognition performance of the method of transferring knowledge in the non-optical domain has been further improved compared with the method based on transferring knowledge in the optical domain, increased by 1.72%, 3.00%, 3.06%, 3.52%, 4.36%, 7.38%, respectively on the small samples datasets of 1/2, 1/3, 1/4, 1/8, 1/16, 1/32, indicating that the higher the similarity of the dataset of the source and target domains, the more it will help improve transfer effect. Compared with the model that has been trained from scratch, the transfer method based on the full-angle data-enhanced SAR data as the source domain dataset has a significant effect on improving the performance of SAR target image recognition, increasing by 2.84%, 6.25%, 5.40%, 12.10%, 13.27%, 19.01%, respectively on the small sample datasets of 1/2, 1/3, 1/4, 1/8, 1/16, 1/32. This significant improvement shows that transfer learning can effectively increase the accuracy of recognition when the data is limited.

#### 4.6.3. Transferring Prior Knowledge of Mixed Optical and Non-Optical Images

In order to further investigate the influence of prior knowledge of the combined optical and non-optical domains as source domain knowledge on SAR target recognition tasks, we have established a complete hybrid dataset based on the NWPU-RESISC45 dataset and 3-class MSTAR dataset rotated at random angles as the source domain dataset, and adopted 10-class MSTAR dataset under SOC is used as the target domain dataset. First, the TAI-SARNET mentioned in this paper is used to train on the hybrid dataset to obtain a pre-trained model. Then, we transfer the weights of the pre-trained model to our target task network and slightly fine tune the initially learned parameters.

From Transfer3 in Figure 8, the method of transferring joint optical and non-optical domain knowledge is better than direct training, and it is similar to the method of transferring only optical domain knowledge, but it is not as effective as the method of transferring only non-optical knowledge. From the above analysis, it can be seen that the knowledge of transferring different source domains using parameter-based migration methods can assist in improving the recognition results of the small samples SAR targets classification tasks, in which the source domain and target domain use homologous imaging mode of data promotion is the best.

### 4.7. Comparison Evaluation

The increasing depth and size of CNNs have brought great challenges to the deployment of deep learning in the terminal equipment, and an efficient SAR targets recognition model needs to consider the number of network parameters and computational complexity while ensuring accuracy. We have tested the representative five classical CNNs and the TAI-SARNET in 10-class MSTAR dataset under SOC, and the experimental results are exhibited in Table 8. As can be viewed from Table 8, LeNet is the network with the least amount of parameters and the smallest storage model size in the classical network, and its network structure is simple, with only 87.96% recognition accuracy in the classification of SAR target images based on the 10 classes task. Although the recognition accuracy of Alexnet, VGG16 and ResNet50 is 93.40%, 90.15% and 89.59%, respectively, these networks are all complex in structure, with huge model parameters and dimensions. MobileNet, which uses deep separable convolution, is a lightweight CNN network focusing on mobile or embedded devices. Although the model size is only 26.1 M, the recognition accuracy is 91.96%. The TAI-SARNET parameters are only 653,818, and the model size is only 5.4 Mb, which is smaller than the simplest structure of LeNet, but the recognition accuracy is the highest at 97.97%. Compared to MobileNet, which is known as lightweight CNN, the parameters and storage model size of our proposed network are only 1/5, but the recognition accuracy is 6.01% higher. Therefore, the TAI-SARNET presented in this paper has reached the level of lightweight CNN in terms of model parameters, storage model size and recognition accuracy.

Similarly, we draw comparisons between the method proposed in this paper and other advanced methods applied to 10 classes of SAR target recognition tasks under SOC as well, and the recognition results are shown in Table 9. The method based on CNN [49] achieves 91.41% recognition accuracy, the method based on sparse representation classifier (SRC) [50] achieves 93.67%, the method based on binary target area analysis [51] achieves 97.72%, the method based on Gabor filter [52] achieves 96.32% and the method based on sparse representation [53] achieves 97.38%. Our method has proved to achieve a recognition accuracy of 97.97%, which fully demonstrates the effectiveness of the TAI-SARNET in SAR target images recognition tasks.

## 5. Conclusions

In view of the difficult problems in the SAR target recognition tasks, an efficient SAR ATR algorithm with transfer learning is proposed. The Atrous-Inception module is designed based on the atrous convolution and the Inception structure. On the one hand, the module can obtain a rich global receptive field, which helps the network to extract detailed information about limited training data, and improves the robustness on small samples datasets. On the other hand, this module controls the augmentation of the number of network parameters and makes the network lightweight. In addition, the use of the BN strategy speeds up the entire training process and effectively alleviates the gradient divergence and overfitting during the training process. Meanwhile, adopting BN strategy to accelerate the whole training process effectively alleviates the gradient divergence and overfitting phenomenon. Finally, transfer learning strategy is used to explore the recognition performance of transferring prior knowledge in the domains of optics, non-optical, joint optic and non-optical to SAR small samples datasets, and the experimental results have shown that the recognition accuracy is considerably improved. For the 10 classes of SAR targets images recognition tasks under SOC, our method has reached 97.97 percent recognition accuracy, and the experimental results have proved the effectiveness of the method. The small sample data has rich information and is closer to the actual application. It is very meaningful to design an efficient CNN model for small sample data. In the future, we are likely to further delve into the application of related methods of transfer learning and few-shot learning in the domain of SAR targets.

## Figures and Tables

**Table 1:** The proposed TAI-SARNET parameters setting.

Description	Output Size	Filter Size/Stride	Parameters
Input	64 × 64 × 1	-	-
Conv1	31 × 31 × 32	3 × 3/2	288
BN	31 × 31 × 32	-	96
Conv2	29 × 29 × 32	3 × 3/1	9216
BN	29 × 29 × 32	-	96
Conv3	29 × 29 × 64	3 × 3/1	18,432
BN	29 × 29 × 64	-	192
Maxpool	14 × 14 × 64	3 × 3/2	-
Conv4	14 × 14 × 80	1 × 1/1	5120
BN	14 × 14 × 80	-	240
Atrous-Inception module	14 × 14 × 96	-	395,616
Atrous-Conv	6 × 6 × 256	3 × 3/1	221,184
BN	6 × 6 × 256	-	768
Global Averagepool	256	-	-
Softmax	10	-	2570
Total			653,818

**Table 2:** 10-class MSTAR dataset configuration under Standard Operating Condition (SOC).

Types	Tops	Train Set			Test Set			Image Size
		Serial	Depression Angle	Number	Serial	Depression Angle	Number	
2S1	Artillery	B_01	17°	299	B_01	15°	274	64 × 64
ZSU234		D_08	17°	299	D_08	15°	274	64 × 64
BRDM2	Truck	E_71	17°	298	E_71	15°	274	64 × 64
BTR60		K10YT	17°	256	K10YT	15°	195	64 × 64
BTR70		C_71	17°	233	C_71	15°	196	64 × 64
BMP2		9563	17°	233	9563	15°	587	64 × 64
					9566			
					C21			
D7		92V	17°	299	92V	15°	274	64 × 64
ZIL131		E_12	17°	299	E_12	15°	274	64 × 64
T62	Tank	A_51	17°	299	A_51	15°	273	64 × 64
T72		132	17°	232	132	15°	582	64 × 64
					812			
					S7			
Total				2747			3203	

**Table 3:** Test result for different dilated rates.

Model	Rate	Accuracy (%)
TAI-SARNET	1	91.72
	2	95.56
	4	**97.97**
	6	88.63
	8	-

**Table 4:** Effect of the number of Atrous-Inception module.

Module	Number	Parameter	Accuracy (%)
Atrous-Inception module	0	221,338	94.28
	1	653,818	**97.97**
	2	1,077,082	96.31
	3	1,500,346	95.59

**Table 5:** The result of different layer merge methods of the Atrous-Inception module.

Module	Type	Parameter	Accuracy (%)
Atrous-Inception module	Concatenate	1,317,370	95.94
	Add	653,818	96.32
	Multiply	653,818	93.35
	Average	653,818	95.32
	Minimum	653,818	93.41
	Maximum	653,818	**97.97**

**Table 6:** Modelling performance of Batch Normalization (BN) strategy.

Model	BN	Accuracy (%)
TAI-SARNET	No	89.60
	Yes	**97.97**

**Table 7:** Confusion Matrix of our method on 10-class MSTAR dataset under SOC.

Types	Artillery		Truck						Tank		Acc (%)
	2S1	ZSU234	BRDM2	BTR60	BTR70	BMP2	D7	ZIL131	T62	T72	
2S1	**274**	0	0	0	0	0	0	0	0	0	100
ZSU234	0	**273**	0	0	0	0	**1**	0	0	0	99.64
BRDM2	**2**	0	**271**	**1**	0	0	0	0	0	0	98.91
BTR60	**4**	0	**3**	**176**	**11**	0	0	**1**	0	0	90.26
BTR70	**1**	0	0	0	**195**	0	0	0	0	0	99.49
BMP2	**5**	0	**2**	**1**	**7**	**558**	0	0	0	**14**	95.06
D7	0	**1**	0	0	0	0	**273**	0	0	0	99.64
ZIL131	0	0	0	0	0	0	**3**	**271**	0	0	98.91
T62	0	0	0	0	0	0	0	0	**273**	0	100
T72	**4**	0	0	0	**1**	**3**	0	0	0	**574**	98.63
Total	97.97										

**Table 8:** Performance comparison with other classical networks.

Method	Parameter	Model Size (Mb)	Accuracy (%)
LeNet	1,134,806	9.1	87.96
AlexNet	21,598,922	172.8	93.40
VGG16	39,928,522	319.5	90.15
ResNet50	23,601,930	189.2	89.59
MobileNet	3,238,538	26.1	91.96
TAI-SARNET	**653,818**	**5.4**	**97.97**

**Table 9:** Performance comparison with other methods.

Method	Accuracy (%)
CNN [49] SRC [50]	91.41 93.67
Binary Target Region [51] Data augmentation with Gabor filter [52] Joint sparse representation [53]	97.72 96.32 97.38
Proposed	**97.97**
